# Vaccination should be everyone's business: Challenges in vaccinating pregnant women against influenza in the Republic of Moldova

**DOI:** 10.1002/ijgo.15896

**Published:** 2024-09-23

**Authors:** Angela K. Shen, Veaceslav Gutu, Alina Druc, Angela Capcelea, Malembe Ebama, Brittany Adams, Asalif Belayneh, Molly Valleau, Angela Paraschiv

**Affiliations:** ^1^ The Task Force for Global Health Atlanta Georgia USA; ^2^ Leonard Davis Institute for Health Economics University of Pennsylvania Philadelphia Pennsylvania USA; ^3^ National Agency for Public Health Chișinău Republic of Moldova; ^4^ UNICEF Chișinău Republic of Moldova; ^5^ US Centers for Disease Control and Prevention Atlanta USA; ^6^ Nicolae Testemițanu State University of Medicine and Pharmacy Chișinău Republic of Moldova

**Keywords:** influenza, maternal immunization, provider recommendation, Republic of Moldova

## Abstract

Obstetricians/gynecologists are not included in annual influenza training and have contraindicated influenza vaccines to pregnant individuals, representing a missed opportunity for a provider recommendation.

Although pregnant women in the Republic of Moldova are targeted for influenza vaccination because of the risk of complications due to illness, fewer than 1% are vaccinated (Table 1). A major driver for hesitancy, as in other countries, is concern for the fetus.^1^ In Moldova, unlike primary care doctors who are trained to provide and promote vaccinations, physician specialists like obstetricians/gynecologists who care for pregnant individuals are not included in annual influenza training, representing a missed opportunity for a provider recommendation.^2^

**TABLE 1:** Doses administered and vaccination coverage for influenza vaccine by priority target group for influenza seasons: 2022–2023, 2023–2024.

Target group	2022–2023	2023–2024
	Estimated coverage (%)	Estimated coverage (%)
Children at high risk	2928/7464 (39.23%)	2986/5016 (59.5%)
Health workers	20 982/36 168 (58.01%)	16 586/36 168 (45.9%)
Pregnant women	203/26 952 (0.75%)	248/26 952 (0.9%)
Adults at high risk (e.g. those living in congregate settings)	187 773/658 082 (28.53%)	174 954/658 082 (26.6%)
Total	218 000/728 666 (29.92%)	194 774/726 218 (26.8%)

*Note*: Estimated coverage is calculated using the doses administered over the planned population. Doses are estimated and planned for by individual health centers who provide denominator estimates for risk groups. These estimates are based on administrative methods. Vaccination coverage for the 2020–2021 and 2021–2022 season for pregnant women was <7%; doses administered in 2020–2021 and 2021–2022 to pregnant women were 1956 and 203, respectively. Notably, COVID‐19 vaccination for pregnant women was also a priority in 2020–2021.

A mixed methods cross‐sectional evaluation of Moldova's national influenza immunization program was conducted from December 4 to 13, 2023, to identify strengths and challenges in administering influenza vaccines to target populations: health workers, adults and children with chronic diseases, and pregnant women.^1^ Influenza vaccines arrived September 2023 and were rapidly distributed to facilities within a few days. Vaccination campaigns were concentrated in October and November 2023, and a new electronic management system (RVC‐19), developed for COVID‐19 campaigns, was used to capture influenza vaccine administration and manage vaccine stock in real time. Patient consent was not required for this evaluation. We obtained approval for this study from the National Committee for Ethical Expertise of Clinical Trial, Republic of Moldova.

Using a modified World Health Organization post‐introduction evaluation tool,^3^ field surveys of immunization program administrators were conducted at national, regional, district, and facility levels. Surveys of the target population and five key informant interviews of national level immunization partners (e.g. UNICEF) were also conducted.

Two districts within each of the three regions (north, center, and south) (Figure S1) were selected for fieldwork. Facilities were selected based on vaccination uptake (fastest or slowest) within the first month of the previous year's campaign and by vaccination coverage (highest or lowest) from previous years. Summary descriptive statistics and a thematic analysis on qualitative data were performed, with results triangulated with a desk review of documents.

A total of 152 interviews were conducted: one national, six districts, 18 health facilities, 18 parents or caregivers, 34 health workers, 44 adults with chronic conditions, 19 pregnant women, and 13 vaccination session observations.

Among the pregnant individuals interviewed, 31.6% (6/19) did not receive a recommendation for influenza vaccination from their provider; notably, one individual received a recommendation from a friend. In contrast, 93.2% (41/44) of adults with chronic conditions and 68.4% (13/19) of children with chronic conditions received provider recommendations. Among the district managers surveyed, 83.3% (5/6) felt that pregnant individuals had only “some acceptance” (1/6) or “no acceptance” (4/6) of influenza vaccination.

Most pregnant women (17/19, 89.5%) declined vaccination, citing fear of adverse effects (9/17, 52.9%), insufficient time or interest (5/17, 29.4%), contraindication by a provider (3/17, 17.6%) or lack of recommendation by a provider (2/17, 11.8/%) (Figure S2). Many of the pregnant individuals surveyed felt the best way to communicate about vaccination was by engaging with their providers in person rather than through media, the internet, or educational materials.

All primary healthcare providers interviewed (34/34) were highly knowledgeable about influenza and had been vaccinated for at least the last two influenza seasons. Among those interviewed, 56% (19/34) supported mandatory vaccination policies for healthcare workers. They emphasized that the main messengers for vaccination are primary care doctors and that specialist doctors are not involved in promoting vaccination. Some interviewees cited that specialists recommended against vaccination to their patients due to contraindications and believed that specialists might be propagating misinformation about the risks and benefits, particularly regarding safety for the fetus. Despite their personal acceptance of influenza vaccination, primary care physicians did express concern about providing strong vaccine recommendations to hesitant individuals or a recommendation that might contradict the advice of specialists. Administrators at national, regional, and facility levels (*n* = 25) and national key informants (*n* = 5) corroborated these claims and confirmed that specialists are not included in annual influenza training and do not receive training around vaccinations in general.

Our evaluation found that pregnant individuals were not consistently advised to receive influenza vaccine by their providers and in some cases were advised against vaccination. Recommendations from a healthcare provider are critical to vaccine acceptance.^2^ Annual trainings, inclusive of a broader range of health professions and provider types (i.e. obstetricians/gynecologists) should emphasize the importance of vaccination as a health worker norm. The antenatal care platform is also a viable early entry point to educate and familiarize pregnant women and their families on life course vaccination^1^ and to introduce the childhood vaccination schedule, which includes a birth dose of hepatitis B vaccine.^4^

Although this evaluation had a limited number of interviews collected through a convenience sample, interviews at all levels corroborated the programmatic limitation that messaging, advocacy, and vaccination across the life course is narrowly concentrated with family doctors.

Engaging existing (i.e. family doctors) and new providers (i.e. specialist doctors) and partners (e.g. organizations supporting antenatal care) to sensitize families and champion vaccines throughout the life course can be a significant step in strengthening the maternal immunization platform and elevating vaccination to become a social norm for current and future vaccines.

## AUTHOR CONTRIBUTIONS

AKS, VG, AD, ME were involved in the design and planning of the study. All authors were involved in the conduct of the study. AKS, ME, BA, AB, and MV were responsible for analysis, and all authors contributed to the writing, review, and approval of the manuscript.

## FUNDING INFORMATION

This evaluation was supported by the Task Force for Global Health under the Partnership for International Vaccine Initiatives (PIVI) Project ID INF‐CDC‐PV5.

## CONFLICT OF INTEREST STATEMENT

The authors declare no conflicts of interest.

## Supporting information


Figure S1.

Figure S2.

## Data Availability

Research data are not shared.
