# Supplementary material for: Vaccination should be everyone's business: Challenges in vaccinating pregnant women against influenza in the Republic of Moldova
Source: Int J Gynaecol Obstet. 2024 Sep 23;168(2):849–51. doi: 10.1002/ijgo.15896 (PMC11726140; doi:10.1002/ijgo.15896)
Supplement: Supplementary file 1 — Figure S1. [file IJGO-168-849-s001.docx]

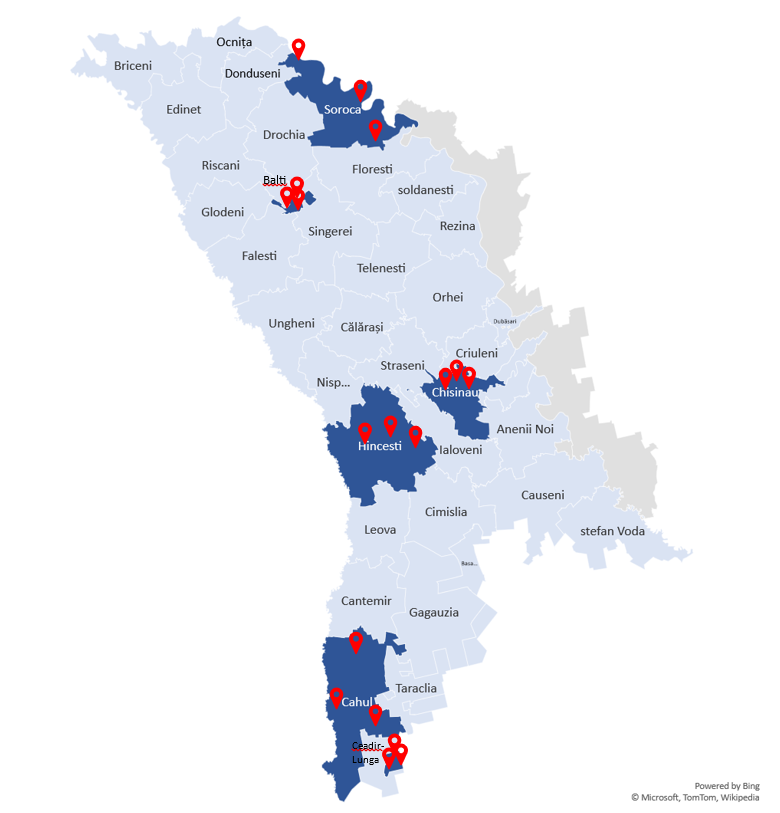

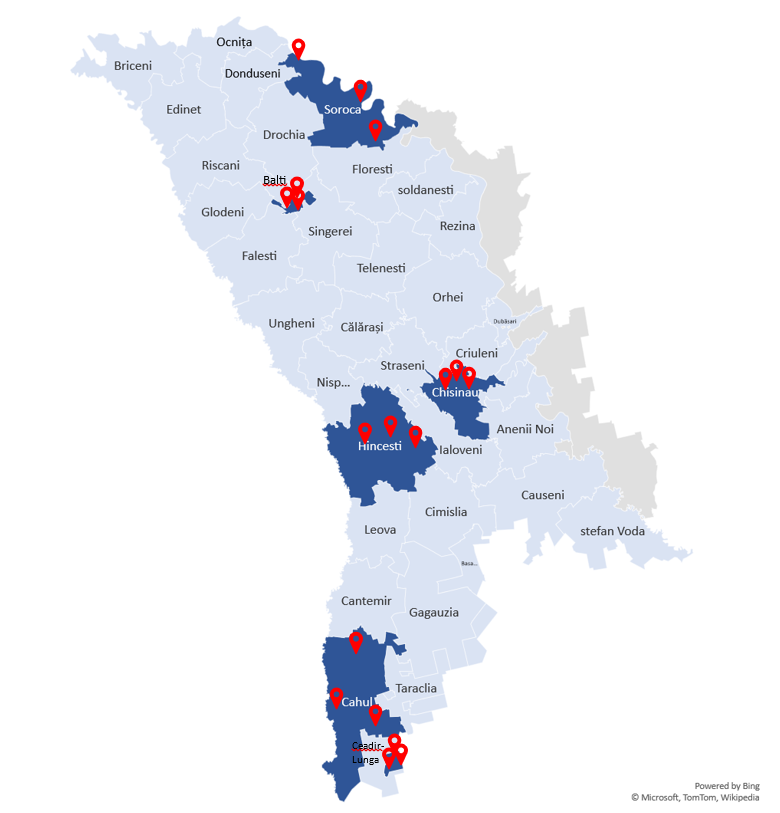
**Supplement Figure 1: Influenza Evaluation Sites in the Republic of Moldova**

**Legend: Red markers represent health centers selected as evaluation sites within six districts or municipalities of Balti, Cahul, Ceadîr-Lunga, Chisinau, Hincesti,** **Soroca**
