# Supplementary material for: Vaccination should be everyone's business: Challenges in vaccinating pregnant women against influenza in the Republic of Moldova
Source: Int J Gynaecol Obstet. 2024 Sep 23;168(2):849–51. doi: 10.1002/ijgo.15896 (PMC11726140; doi:10.1002/ijgo.15896)
Supplement: Supplementary file 2 — Figure S2. [file IJGO-168-849-s002.docx]

**Supplemental Figure 2: Primary Reasons for Refusing Vaccination for Pregnant persons and All Target Groups**


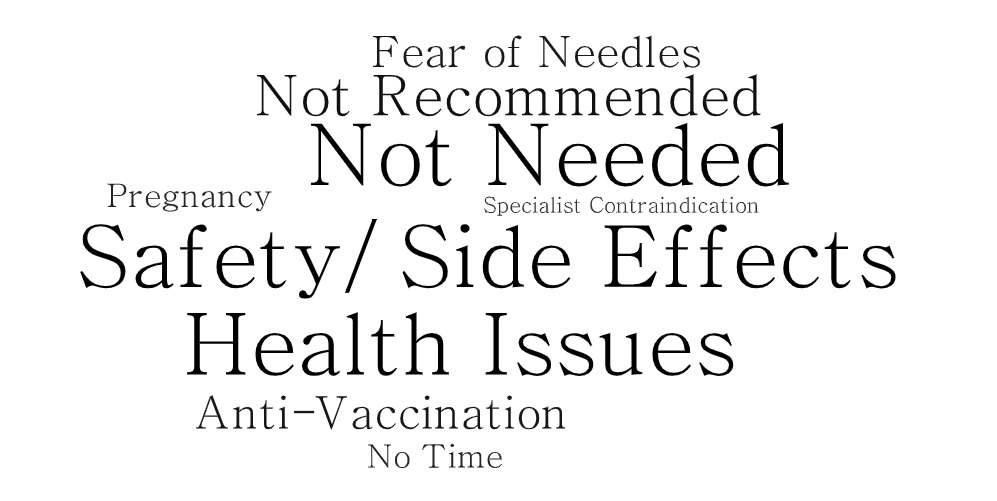


Note: Pregnant persons, n=17


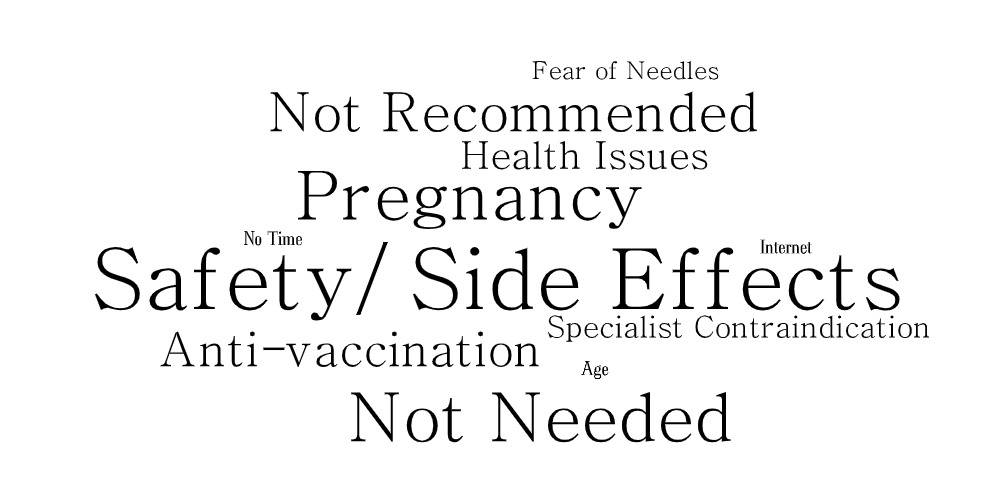


Note: All Target Groups (Adults and Children with Chronic Disease and Pregnant persons), n=37. Health workers did not refuse vaccination.
